# Optimal Design of a Polyaniline-Coated Surface Acoustic Wave Based Humidity Sensor

**DOI:** 10.3390/s131216816

**Published:** 2013-12-05

**Authors:** Wen Wang, Xiao Xie, Shitang He

**Affiliations:** State Key Laboratory of Acoustics, Institute of Acoustics, Chinese Academy of Sciences, No.21, BeiSiHuan West Road, Beijing, China; E-Mails: xz7807330966@live.cn (X.X.); heshitang@mail.ioa.ac.cn (S.H.)

**Keywords:** Al/Au electrodes, COM model, humidity sensor, oscillator, polyaniline, resonator

## Abstract

This paper presents an optimal design for a new humidity sensor composed of a dual-resonator oscillator configuration with an operation frequency of 300 MHz, and a polyaniline (PANI) coating deposited along the resonation cavity of the sensing device. To improve the corrosion resistance of the sensor chip, Al/Au electrodes were used to form the SAW resonator. Prior to device fabrication, the coupling of modes (COM) model was used for the performance prediction and optimal design parameters determination. Two SAW resonators with Al/Au electrodes were fabricated on an ST-X quartz substrate, and used as the frequency control element in the feedback path of an oscillator circuit. A PANI thin coating was deposited onto the resonator cavity of the sensing device by a spinning approach as the sensor material for relative humidity (RH) detection. High detection sensitivity, quick response, good repeatability and stability were observed from the sensor experiments at room temperature.

## 1. Introduction

**Figure 1.**
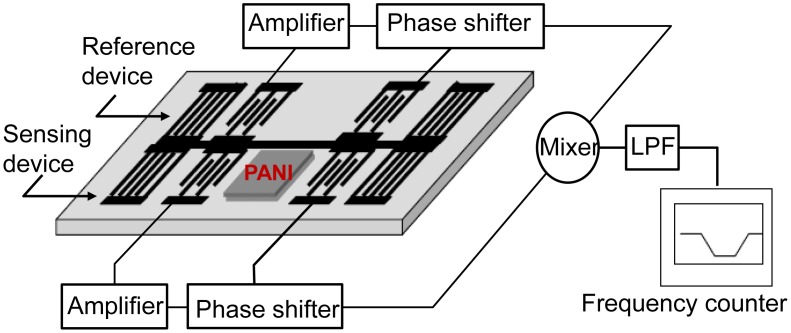
The schematic and principle of the SAW-based humidity sensor.

Humidity detection has been attracting increasing interest over the past years in the fields of industrial and agricultural production, food storage, meteorology, environment protection, *etc.* [1]. Recently, surface acoustic wave (SAW)-based humidity sensors have attracted much attention since they exhibit the advantages of very fast response (several seconds), high sensitivity, small size, integrated electronic circuitry, and easy to realize wireless communication over the current impedance-type or capacitance type humidity sensors [2–4], and also the optical sensors coated with chiral sculptured thin films or thin dielectric waveguide [5,6]. The schematic and working principle of a typical SAW-based humidity sensor with a dual-oscillator configuration is shown in Figure 1, where the SAW devices are used as frequency control elements in the feedback path of an oscillator circuit. A sensitive interface allowing analytes to be sorbed onto the device surface was deposited along the acoustic wave propagation path of the sensing device. The physical adsorption between the sensing film and the target water vapor species modulates the phase velocity of the SAW propagating along the SAW device, and the target relative humidity can be characterized by the oscillation frequency shift.

However, even though there are attractive reports about SAW-based humidity sensors, they still suffer from poor corrosion resistance of the sensor chip itself because of their use of Al electrodes. Additionally, deficiencies of the optimized design parameter extraction of the SAW devices leads to poor oscillator frequency stability, and thus directly affects the limit of detection and stability of the gas sensor. Up to now, two types of SAW device configuration were reported to be used as the feedback element of the oscillator for gas sensing [7]. One is delay line structured by two interdigital transducers (IDTs) and a delay path, that can provide enough sensitive film deposition area but relatively low Q-value and larger insertion losses affecting the frequency stability of the oscillator. The other is a resonator configuration composed of two reflectors and the adjacent transducers. The two-port SAW resonators with aluminum (Al) electrodes are widely used as the frequency feedback element due to their high electrical quality factor (Q) value and low insertion loss over the delay line patterns, resulting in excellent noise immunity and high measurement resolution and accuracy [8–10]. However, such sensor systems still suffer from a major problem, in that if the sensors are operated in chemically reactive gas-phase environments, the Al electrode structure of the sensor device is easily attacked by the detected gas or gas mixture which forms corrosive acids or bases with the humidity of the ambient air. The problem is further aggravated if the sensing polymer film on the device surface greatly increases the amount of adsorbed agent and moisture coming in contact with the electrode structure. As a result of that, the sensor performance degrades and the device electrode structure is easily destroyed. The solution to such problems is the implementation of SAW resonant devices using corrosion-proof electrodes of gold (Au) or platinum (Pt). Very impressive results on low-loss resonator filters using heavy metals in their electrode pattern, including Au, have been recently reported [11]. Unfortunately, these devices use the shear-horizontal leaky SAW mode, which does not operate so well with the soft polymer films required for high gas sensitivity, as the Rayleigh SAW (RSAW) mode does [12]. Recently, a Au-RSAW two-port SAW resonator, operating at 433 MHz with a typical loaded Q as high as 5,000 and insertion loss in the −8 to −10 dB range in the uncoated state have been reported for gas sensing [7,13]. However, except for a substantial increase in production cost, much higher velocity perturbation by Au may result in serious distortion of the frequency and phase responses, and also, the much larger density of Au compared to Al induces strong excitation of a parasitic surface slimming bulk wave (SSBW) mode. To solve such issues, Wang *et al*. presented a new design of a SAW device using a dual-layers electrode structure of Al and a very thin Au film on top of the Al [14]. Liu *et al*. characterized the electromechanical coupling factor (*K*^2^%) and reflection coefficient of the Al/Au electrodes by using the theory of acoustic propagation and variational principle of short-circuited grating [15]. The Al/Au resonators feature insertion losses and loaded Q values comparable with those of SAW resonators with Al or Au metallization, currently used in gas sensor systems. First, a thin Au layer not only reduces the cost, but also prevents the attack from the measured gases on the Al electrode, and also, the perturbation from the electrode on the SAW velocity and electromechanical coupling factor is reduced significantly because of the very thick Al film design, leading to performance improvements and technique simplification. Hence, the first purpose of this paper is to develop a two-port SAW resonator with Al/Au electrodes and excellent performance features like lower insertion loss, high Q-values and single-mode characteristic for humidity sensing. Prior to device fabrication, the coupling of modes (COM) was referred to the SAW device for performance prediction and optimal design parameters extraction.

The second aim of this paper is to present a new SAW humidity sensor using the developed resonator-oscillator with excellent frequency stability as the sensor element. In this paper, a PANI thin film was utilized as the sensor material for relative humidity detection. As a conducting polymer, it has received a great deal of attention owing to its simple synthesis, good environmental stability, ability to be doped with protonic acids and moderately high electrical conductivity [16]. In the process of humidity detection, the water molecules or moisture adsorption modulate the conductivity of the PANI, resulting in a clear perturbation of SAW propagation. Using the differential oscillation structure as shown in Figure 1, the mixed frequency signal was used to characterize the relative humidity (RH). The performance features of sensitivity, stability, and repeatability of the present 300 MHz SAW-based humidity sensor were evaluated experimentally.

## 2. COM Simulation on SAW Devices

**Figure 2.**
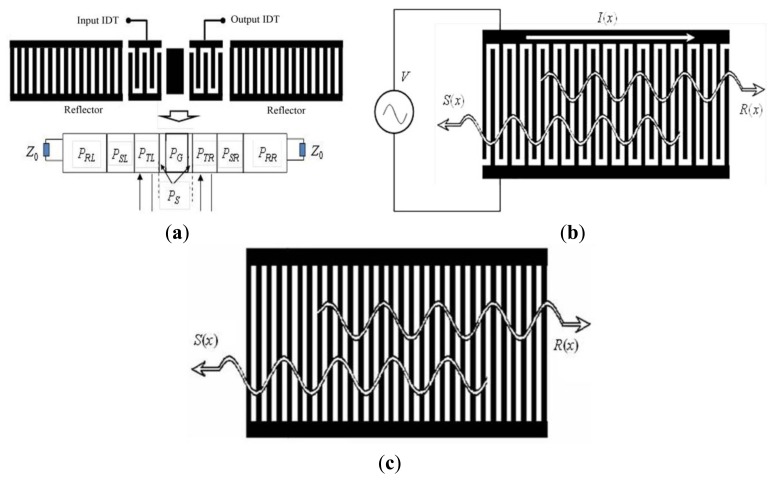
(**a**) SAW resonator structure and the corresponding P matrices; (**b**) COM model for IDTs; (**c**) COM model for reflector.

In this section, the COM model was referred to for optimal simulation of the two-port SAW resonator with Al/Au electrodes for use as a humidity sensor. COM modeling is a very efficient technique developed for the analysis of the SAW device. Plessky [17] reviewed COM equations for SAW devices where the acoustic waves propagating in the forward and reverse direction and incorporated their coupling interaction. For optimal simulation on the SAW resonator configuration as shown in Figure 2, a COM model was used to analyze the IDTs and reflectors, respectively. By using the extracted mixed P-matrix of the IDTs, reflectors, coating area in the resonance cavity made by metal thin film for PANI deposition, and the gaps between the IDTs and reflectors (Figure 2a), the device admittance matrix Y can be deduced, and hence, the frequency response, *S_12_*, is obtained.

### 2.1. COM Analysis for IDTs

The COM equation for IDT deals with acoustic waves propagating in the forward and reverse directions and incorporates their coupling interaction, as shown in Figure 2b. *R(x)* and *S(x)* are slowly varying two acoustic wave amplitudes. Then, the 3 × 3 *P*-matrix representation is used to present the solutions to the COM equations (Equation (1)) [17]:

(1) $$\{\begin{array}{l} dR(x)/dx=-i\delta R(x)+i\kappa S(x)+i\alpha V\\ dS(x)/dx=-i\kappa^{*}R(x)+i\delta S(x)-i\alpha^{*}V\\ dI(x)/dx=-2i\alpha^{*}R(x)-2i\alpha S(x)+i\omega CV \end{array}$$

where *δ* is the coupling coefficient, *κ* is the electrode reflection coefficient, *α* is the transduction coefficient, *C* and *ω* are unit capacity and angular frequency respectively. The three equations in the COM modeling can be integrated, so that all parameters in the *P*-matrix can be evaluated as:

(2) $$\left[\begin{array}{c} S(0)\\ R(L)\\ I(0) \end{array}\right]=[P]\left[\begin{array}{c} R(0)\\ S(L)\\ V \end{array}\right]=\left[\begin{array}{ccc} P_{11} & P_{12} & P_{13}\\ P_{21} & P_{22} & P_{23}\\ P_{31} & P_{32} & P_{33} \end{array}\right]\;\left[\begin{array}{c} R(0)\\ S(L)\\ V \end{array}\right]$$

where, L denotes the transducer length.

### 2.2. COM Analysis for Reflectors

As shown in Figure 1c, the COM equations for shorted grating reflectors are:

(3) $$\{\begin{array}{c} \frac{dR(x)}{dx}=-i\delta_{s}R(x)+i\kappa_{s}S(x)\\ \frac{dS(x)}{dx}=-i{\kappa_{s}}^{*}R(x)+i\delta_{s}S(x) \end{array}$$

where, *δ_s_* and *κ_s_* are coupling coefficient and reflection coefficient respectively. The COM equations for reflectors are as:

(4) $$\left[\begin{array}{c} S(0)\\ R(L) \end{array}\right]=\left[\begin{array}{cc} P_{\text{ref}11} & P_{\text{ref}12}\\ P_{\text{ref}21} & P_{\text{ref}22} \end{array}\right]\;\left[\begin{array}{c} R(0)\\ S(L) \end{array}\right]$$

*L_R_* denotes the length of the reflectors.

### 2.3. COM Simulation Results and Discussion

Using cascading [18] P matrices (*P_RL_*) from the left reflector in Figure 2a, the gap between the left reflector and the IDT (*P_SL_*), IDT (*P_TL_*), the gap between the IDT and the coating area (*P_S_*), and the coating area (*P_G_*), the P matrix for the left components is obtained as *P_LIDT_* by using the cascading relationships. Similarly, the right counterpart is cascaded as *P_RIDT_*. Then, by cascading the *P_LIDT_* and *P_RIDT_*, the admittance Y-matrix can be expressed by:

(5) $$Y=\left[\begin{array}{cc} y_{11} & y_{12}\\ y_{21} & y_{22} \end{array}\right]$$

where:

(6) $$\begin{array}{c} y_{11}={\text{P}}_{\text{LIDT}33}+\frac{{\text{P}}_{\text{RIDT}11}{\text{P}}_{\text{LIDT}32}P_{\text{LIDT}23}}{1-{\text{P}}_{\text{RIDT}11}P_{\text{LIDT}22}},y_{12}=\frac{{\text{P}}_{\text{RIDT}13}P_{\text{LIDT}32}}{1-P_{\text{RIDT}11}P_{\text{LIDT}22}}\\ y_{21}=\frac{P_{\text{RIDT}31}P_{\text{LIDT}23}}{1-P_{\text{RIDT}11}P_{\text{LIDT}22}},y_{22}=P_{\text{RIDT}33}+\frac{P_{\text{LIDT}22}P_{\text{RIDT}13}P_{\text{RIDT}31}}{1-P_{\text{RIDT}11}P_{\text{LIDT}22}}. \end{array}$$

Using the admittance matrix solution, the frequency response S_21_ and the phase response φ of the two-port SAW resonator can be deduced by:

(7) S21=−2y12GinGout(Gin+y11)(Gout+y22)−y12y21φ=atan(Im(S21)/Re(S21))×180/π

where, *G_in_* and *G_out_* are input and output impedance respectively.

To pursue low insertion loss and single-mode for the resonator, the gap between the reflectors and the IDT, as well as the gap between the IDT and coating area and the coating length, can be adjusted strategically. COM simulation was performed for the SAW resonator with Al/Au electrodes in terms of different values of coating area length to extract the optimal design parameters. Additionally, the main parameters in the COM simulation are the reflectivity (*κ*) and acoustic wave velocity shift (Δ*V*/*V*_0_, *V*_0_ is the acoustic wave velocity in case of free surface) in the metallic surface. The terms κ and Δ*V*/*V*_0_ can be characterized by [19]:

(8) $$\begin{array}{c} \kappa =R_{m}\frac{h}{\lambda }+R_{e}\\ \frac{\Delta V}{V_{0}}=ϒm\frac{h}{\lambda }+ϒe \end{array}$$

where *R_m_* and *γ_m_* show the contribution of the constants of the mechanical mass and stress on reflectivity and acoustic wave velocity change, and the *R_e_* and *γ_e_* are the constants of the electrical shifting effect by the electrode deposition toward reflectivity and acoustic wave velocity change, respectively. *h* is the electrode thickness, and the *λ* is the acoustic wave wavelength.

**Figure 3.**
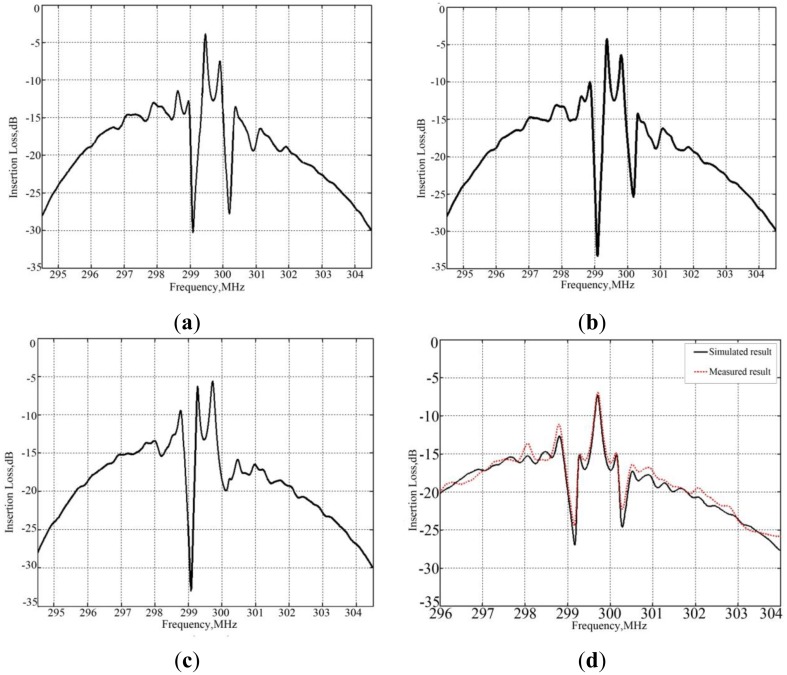
Simulated frequency response of SAW resonator with different coating area length (**a**) 145.0 λ; (**b**) 145.1 λ; (**c**) 145.2 λ; (**d**) 145.3 λ (dotted line: measured data).

Considering the bi-layer electrodes, the reflectivity per electrode can be obtained by the sum of the reflectivity of each of the Al and Au layers. The contribution to the acoustic wave velocity shift from the Al and Au deposition was considered similar to the case of the reflectivity. ST-X quartz was used as the substrate for its excellent room temperature stability. The parameters for the device structure are listed in Table 1. Figure 3 shows the coating area length dependence of the frequency response of the resonator. To obtain a single steep resonance peak, 145.3 λ of coating area was chosen in the design in case Al/Au thicknesses of 100 nm/20 nm are applied. Moreover, very low insertion loss of 5.3 dB and high Q factor of ∼3,000 were obtained.

## 3. Technique Realization

### 3.1. SAW Device Fabrication

**Figure 4.**
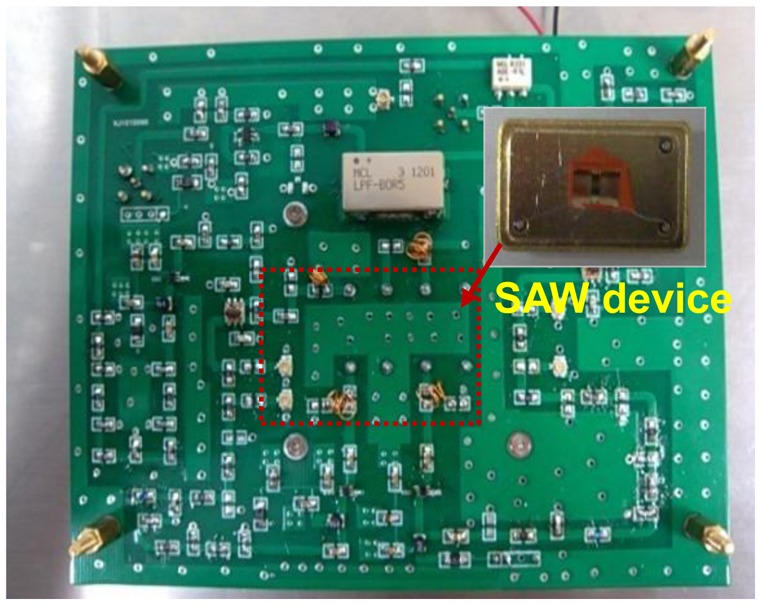
The PCB of SAW oscillation circuit.

Based on the extracted design parameters from the COM modeling, a Al/Au-strip two-port SAW resonator with operation frequency of 300 MHz was reproducibly fabricated, where aluminum with a thickness of 100 nm and Au with a thickness of 20 nm were deposited onto a temperature-compensated ST-X quartz wafer by utilizing the lift-off photolithographic process. The metal coating area length between the IDTs of the resonator was designed to 145.3 λ. In addition, the gap between the IDT and adjacent reflector was set to 0.75 λ. The fabricated SAW device is shown in the inset of Figure 4. Then, referring to the network analyzer, the fabricated SAW resonators were characterized, and a very low insertion loss of 5.3 dB, high unloaded Q-value of ∼3,000, and single-mode were obtained, as shown in Figure 3d. Also, the measured frequency response agrees well with the COM simulated result, demonstrating the good validity of the COM model.

### 3.2. SAW Oscillator

**Figure 5.**
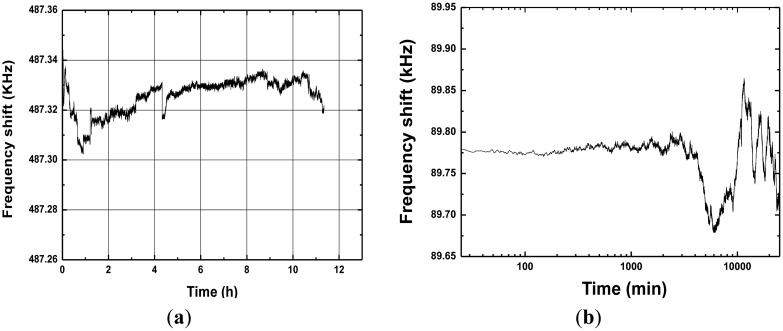
(**a**) The measured medium-term, and (**b**) long-term frequency stability of the developed SAW oscillator.

Next, the fabricated SAW device chip was loaded into a standard metal base (see the inset of Figure 5). As the oscillator feedback, the launching and read transducers of the fabricated SAW resonators were connected by an oscillator circuit which was made of discrete elements (amplifier with a gain of 25 dB, phase shifter, mixer and LPF and so on) on a printed circuit board (PCB) as shown in Figure 4. The output of the amplifier was mixed in order to obtain a difference frequency in the MHz range. This technique allows us to reduce the influence of the thermal expansion of the substrate. The output of the oscillator was monitored and recorded by the computer in real-time. Then, an experiment was performed to evaluate the frequency stability of the fabricated SAW oscillator using the programmable frequency counter at room temperature (20 °C). The oscillation was also modulated at the frequency point with lowest insertion loss by a strategically phase modulation [14]. The measured frequency stability is shown in Figure 5. The typical medium-term frequency stability in hours was measured as ±15 Hz/h (0.1 ppm), as shown in Figure 5a. Moreover, long-term frequency stability of the oscillator used the fabricated SAW resonator with Al/Au electrodes was tested at exposed environment as shown in Figure 5b. Due to the excellent corrosion resistance of the sensor chip itself, very good long-term frequency stability of ±80 Hz/d (0.5 ppm) was obtained. Excellent frequency stability observed from the fabricated oscillator is very significant for performance improvement of SAW sensor.

### 3.3. Sensing Layer Depostion

In this paper, PANI was considered as the sensor material for humidity detection. It is an environmentally stable conducting polymer with excellent electrical, magnetic and optical properties. It has attracted considerable attention over the past 10 years and is generally regarded as a conducting polymer with very high potential in commercial applications as humidity sensors [20–22]. In general, the oxidation level of a polyaniline synthesized by either a chemical or electrochemical method can be described by the following molecular formula [22]:


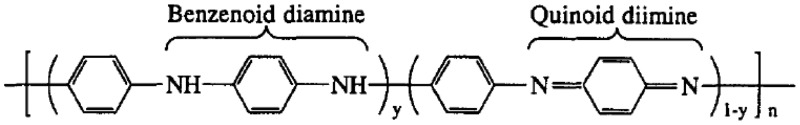


where, y = 1, 0.5 and 0, the corresponding polymers are the fully reduced polyaniline (benzenoid diamine), the half oxidized polyaniline (emeraldine), and the fully oxidized polyaniline (quinoid diimine), respectively. The emeraldine form of polyaniline showed the highest electrical conductivity after it had been doped with a protonic acid, and it is very promising candidater for humidty detection. Such a polymer was synthesized by oxidative polymerization of aniline in aqueous hydrochloric acid solution at room temperature using potassium dichromate as an oxidant [22]. Then, the prepared PANI was deposited onto the coating area of the sensing SAW resonator in Figure 1 by using the solvent-evaporation method. The effectiveness of the solvent evaporation method to produce a stable polymer coating depends on the election of the solvent type [23]. In our experiment, chloroform was used as the solvent. Before the deposition of the PANI film on the SAW device surface, the quartz surface was cleaned of any contaminants by a routine cleaning procedure involving rinsing in piranha solution (V(H_2_SO_4_):V(H_2_O_2_) = 3:1), a DI water rinse and drying by N_2_. Then, 0.1 mL of 0.8 g/L PANI/chloroform solution was deposited on the cleaned quartz surface 10 consecutive times, and then, the PANI-coated SAW device was baked for 3 h at a temperature of 110 °C. To monitor the film deposition, the sensor response for each time was recorded by the frequency counter (Proteck C3100, Seoul, Korea). Making use of the Sauerbreg law, the SXFA thickness was evaluated to be 20 nm.

## 4. Sensor Experiments

### 4.1. Experimental Setup

**Figure 6.**
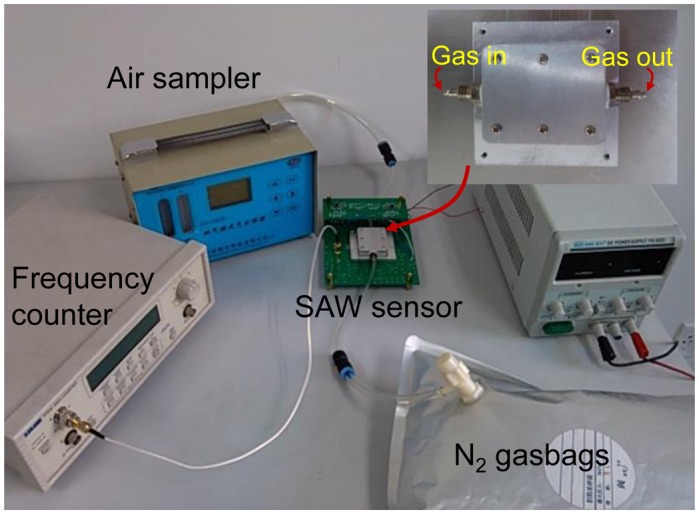
The experimental setup of the SAW humidity sensor.

The experimental setup was composed of the developed SAW sensor system, programmable frequency counter, hygrometer, thermometer, double road air sampler, and N_2_ gasbags, as shown in Figure 6. The developed PANI-coated sensor was kept inside a closed chamber with two separate gas channels (inset of Figure 6) whose cover was made from aluminum to serve as a common ground for the electronic system, and connected to the electronic oscillator circuits.

A frequency counter (TTi, 930) was used to monitor the output signals from the sensor, and connected to a microcomputer system through a GPIB interface board which enabled, using the Timeview software (which was supplied with the frequency counter), collecting and saving the data in a text file for future analysis. A small nebulizer and a double road air sampler are connected to a dry nitrogen airbags, were used to generate humid air to control the RH inside the chamber. RH and temperature were measured using a commercial precision hygrometer and thermometer.

### 4.2. Sensor Response

**Figure 7.**
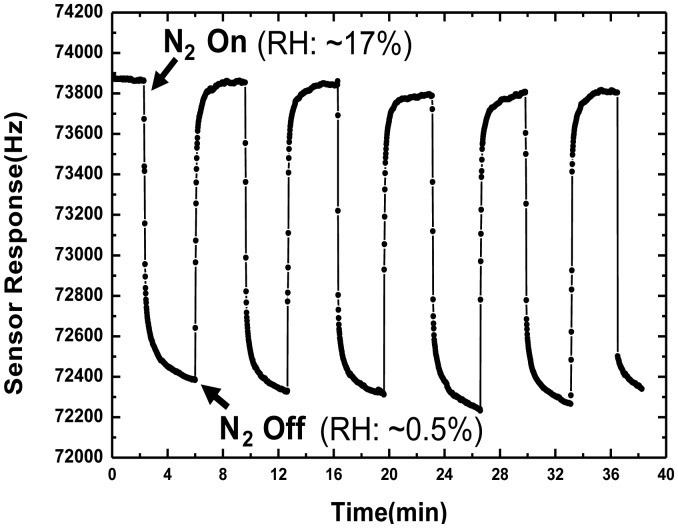
Frequency shift as a function of time, while the RH was changing in the range of 0%–17% (adsorption) and 17%–0% (desorption).

Next, the sensor was characterized regarding repeatability, and frequency shift sensitivity to RH. The repeatability test of the sensor was performed by six consecutive 240-second on-off exposures to 17% of RH at 20 °C, as shown in Figure 7. It can be noted that four gas exposures show a good reproducible run. When the dry N_2_ gas was infused (on state) into the chamber, that is, the RH inside the chamber was reduced to a very low level (∼0.5%, measured by the hygrometer), the frequency response showed a rapid decease and finally reached its saturation value in 6 s. A larger sensor response of around 1,500 Hz was obtained in the RH range of ∼0.5%–17%. When the gas was in the off state, the RH inside the chamber was quickly restored to ∼17%, and the frequency response rapidly fell to its initial value. From this promising result, excellent short term repeatability and fast response towards RH were observed from our developed SAW humidity sensor. Then, the developed SAW humidity sensor was exposed to various RH levels. The RH inside the chamber was controlled by the ventilation of nitrogen and nebulizer, and monitored by the hygrometer.

**Figure 8.**
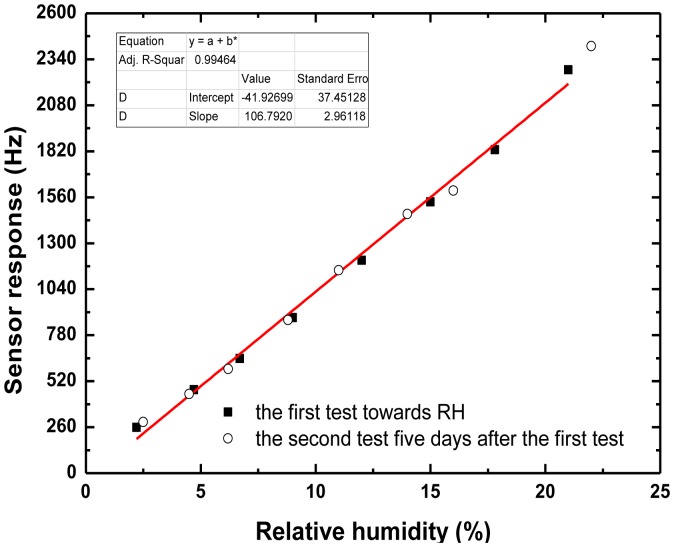
The sensor response towards low humidity and sensor stability testing.

Figure 8 shows the frequency response as a function of low RH. In the range 0%–20% RH, the sensor response exhibits ideal linearity. And the sensitivity was evaluated as 106 Hz/%RH. Also, from Figure 8, a sensor response of ∼260 Hz occurs at a RH of 2.2%. It means lower threshold detection limit will be achieved using the present oscillator with superior frequency stability. According to the International Union of Pure and Applied Chemistry (IUPAC), detection limits are calculated as the lowest concentration of an analyte giving a signal of three-times the medium-term frequency stability of the sensor system. In our sensor, it means the lower detection limit of 0.4% is possible owing to the excellent medium-term frequency stability of the oscillator (±15 Hz/h). The measured data is better than the reported values from similar sensor structures [1]. Moreover, due to the good corrosion resistance of the sensor chip using the Al/Au electrodes, almost no obvious shift was found in the second test five days after the first sensor experiment, as shown in Figure 8, indicating that the sensor performance is very stable.

**Figure 9.**
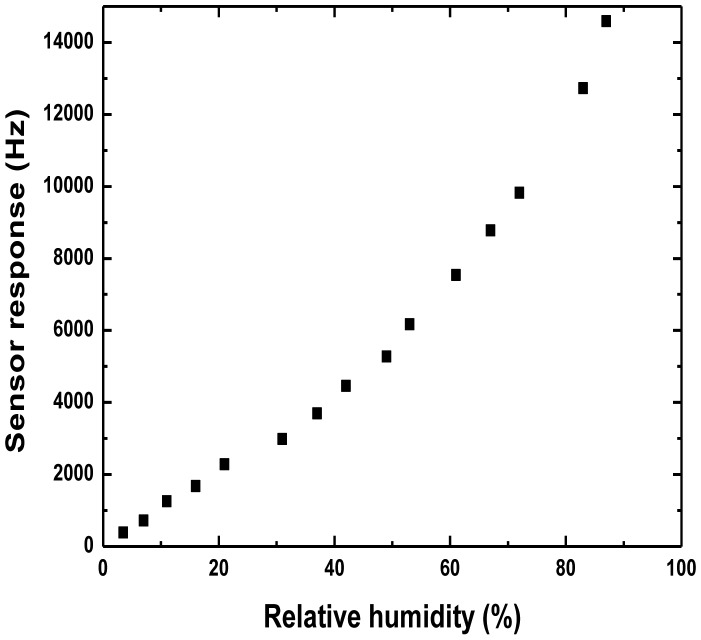
Sensor response towards larger range of RH (0%–87%).

However, a more complex situation was observed in a larger range of RH, as shown in Figure 9, from the measured data, it shows that the behavior is linear in the range 0%–20% RH, becomes increasingly nonlinear for higher values. The reason may be, at low humidity, only the conductivity changes induced by the water vapour adsorption in the PANI was considered as the main sensor response mechanism; but at high humidity, in addition to the conductivity changes from the water vapor adsorption, the viscoelastic effect of PANI itself will also lead to greater disturbance of the surface acoustic wave propagation, resulting in a substantial increase in the sensor response, and leading to nonlinear characteristics of the sensor response. Our future work will examine this point.

## 5. Conclusions

An optimal design was developed fot a new PANI-coated SAW-based humidity sensor, that is, a two-port SAW resonator with a Al/Au electrode was designed as the feedback element of the oscillator. Prior to fabrication, optimal design parameters were extracted by using the COM model. Using PANI as the sensitive material for humidity detection, a 300 MH SAW-based humidity sensor was developed, and the sensor performance was characterized in detail. Ideal linearity was observed in low humidity detection, and also, very good stability was obtained owing to the good corrosion resistance of the sensor chip using the Al/Au electrodes.

## Figures and Tables

**Table 1.** Simulation parameters for SAW resonator with Al/Au electrode structure.

	**Parameters**		**Values**
**Al**	Operation frequency (MHz)		300
	IDT length (λ)		41
	Reflector length (λ)		300
	aperture (λ)		200
	Reflectivity properties of Al electrode	*R_m_*	−0.71
		*R_e_*	−0.00057
	Acoustic velocity propagation of Al electrode	*γ_m_*	−0.17
		*γ_e_*	−0.00058

**Au**	Wavelength (λ: μm)		10.5
	Gap between the reflectors and IDT (λ)		0.75
	Length of the coating area (λ)		145.3
	Gap between the IDT and coating area (λ)		10
	Reflectivity properties of Au electrode	*R_m_*	1.51
		*R_e_*	−0.00057
	Acoustic velocity propagation of Au electrode	*γ_m_*	−4.01
		*γ_e_*	−0.00058
